# Honey Bee Colony Health in Thiamethoxam‐Treated Sugar Beet Fields: A Field‐Based Case Study

**DOI:** 10.1002/ece3.72767

**Published:** 2025-12-23

**Authors:** Richard Odemer, Stefan Berg, Jens Pistorius, Ingrid Illies

**Affiliations:** ^1^ Julius Kühn‐Institut (JKI) – Federal Research Centre for Cultivated Plants Institute for Bee Protection Braunschweig Germany; ^2^ Bavarian State Institute for Viticulture and Horticulture Institute for Bee Research and Beekeeping (IBI) Veitshöchheim Germany

**Keywords:** Field‐realistic pesticide risk evaluation, honey bee colony resilience, pollinator health in agricultural landscapes, sugar beet neonicotinoid impacts, thiamethoxam exposure assessment

## Abstract

Emergency use of thiamethoxam seed treatments in sugar beet was approved in Germany in 2021, despite EU restrictions on neonicotinoids because of pollinator risks. During the field experiment underlying this case study, residues in bee‐relevant matrices were detected only at very low levels, and conservative exposure modeling indicated no acute or chronic concern for honey bees. Building on these exposure findings, the present analysis examined whether such exposures translated into measurable effects on honey bee colony performance. In Experiment 1, colony development was monitored at two geographically distinct sites across the 2021 sugar beet season. Colonies at both sites exhibited strong seasonal growth. Generalized linear mixed models detected no consistent adverse effects of thiamethoxam treatment on either adult bee populations or brood cell numbers. Although temporal fluctuations and site‐specific variability were evident, treatment effects were not statistically supported, highlighting the importance of multi‐site approaches when assessing pesticide impacts and the need for continued multi‐year evidence under diverse environmental conditions. In Experiment 2, survival of individual workers was evaluated using free‐flying mini‐hives. Mixed‐effects Cox modeling, which accounted for colony variance, found no significant differences in worker longevity between treated and control groups. This indicates no evidence for reduced worker survival under a field‐relevant neonicotinoid exposure scenario. Together, these two complementary experimental approaches show that thiamethoxam seed treatments in sugar beet did not cause consistent adverse effects on honey bee colonies under the tested agricultural conditions. By integrating residue analyses, statistical modeling, and colony‐level monitoring, the study provides ecologically relevant evidence that current agricultural practices with thiamethoxam in sugar beet pose a low apparent risk to honey bee colony health, while underscoring the value of longer‐term and broader‐scale field evaluations.

## Graphical abstract

Using multi‐site field trials during Germany's 2021 emergency authorization of thiamethoxam‐treated sugar beet, we combined colony demography and residue analyses with complementary worker survival assays to characterize honey bee exposure under realistic conditions. Across sites, no consistent adverse effects were observed; a late‐season reduction in adult bee numbers at the JKI site numerically exceeded EFSA's 2023 SPG threshold but was not replicated. Residue data indicated minimal bee exposure within sugar beet production systems.

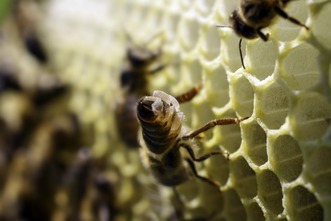

## 1 Introduction

Honey bees ( *Apis mellifera* L.) are key pollinators in agricultural ecosystems and natural landscapes, playing a vital role in biodiversity conservation and global food security. Their health and survival are essential for sustaining ecosystems and agricultural productivity. Yet, widespread reports of honey bee population declines have raised concerns about the impact of modern agricultural practices, particularly pesticide use (Insolia et al. 2022; Siviter and Muth 2020). Among these stressors, neonicotinoid insecticides such as thiamethoxam (TMX) and its metabolite clothianidin (CLO) have been widely applied because of their high efficacy and systemic properties (Giorio et al. 2021; Thompson et al. 2021). Although effective in pest management, their systemic nature means residues can occur in nectar, pollen, and guttation fluids, thereby exposing non‐target organisms (Woodcock et al. 2021).

Concerns over unintended side effects of neonicotinoids on bees have grown steadily during the past two decades. Although initially valued for their relatively low vertebrate toxicity, their primary mode of action—targeting the insect nervous system—renders them intrinsically hazardous to pollinators. Laboratory and semi‐field studies have demonstrated sublethal effects on foraging, navigation, learning, immune function, and reproduction, even at low doses (Tsvetkov and Zayed 2021; Samson‐Robert et al. 2017). Such findings informed the European Food Safety Authority (EFSA) risk assessments and led to restrictions on outdoor use of TMX and CLO in flowering crops (EFSA 2018a, 2018b). Nonetheless, several Member States, including Germany, granted emergency authorizations for TMX seed treatments in sugar beet, highlighting the ongoing tension between agronomic necessity and pollinator safety (Odemer et al. 2023).

Sugar beet ( *Beta vulgaris* ) does not flower in its cultivation year and is therefore often assumed to pose limited direct risk to pollinators. However, secondary exposure pathways remain relevant, including residues in flowering weeds, succeeding crops, or environmental matrices such as soil and nesting substrates (Woodcock et al. 2021; Krupke et al. 2012).

As part of the same 2021 field experiment reported in Odemer et al. (2023), very low residues of TMX and CLO were detected in weed pollen and in mud walls of *Osmia* nesting cavities placed adjacent to treated sugar beet fields. These findings confirmed exposure pathways beyond the treated crop itself. To evaluate potential implications, risk modeling was performed using the U.S. EPA's BeeREX approach (U.S. Environmental Protection Agency 2014), which is validated for seed treatments and more appropriate in this context than the commonly applied Hazard Quotient (HQ) method (Thompson 2021). The resulting risk quotients indicated no acute or chronic concern for honey bees at the residue levels observed.

Because the use of TMX in sugar beet was granted under EU emergency approval, it remains essential to verify whether these exposure conditions translate into colony‐level effects. Therefore, in the present study, we assessed colony development and worker survival outcomes under the same exposure conditions, interpreted in the context of EFSA's Specific Protection Goals (SPGs) for honey bees (EFSA 2023).

Laboratory experiments provide valuable insights into mechanisms of toxicity but often isolate stressors and apply concentrations exceeding those found in agricultural landscapes, which can exaggerate risk estimates (Henry et al. 2015; Alberoni et al. 2021). Field studies, by contrast, incorporate the buffering capacity of colonies and the complexity of real‐world conditions, including forage diversity, weather, and pathogen pressures (Ulgezen et al. 2021; Harwood and Dolezal 2020; Dively et al. 2015). Yet, relatively few field trials have investigated neonicotinoid seed treatments in sugar beet, and even fewer have directly linked residue findings to colony‐level endpoints (Carlson et al. 2022; Thompson et al. 2021). This gap limits the robustness of pollinator risk assessments.

**FIGURE 1:**
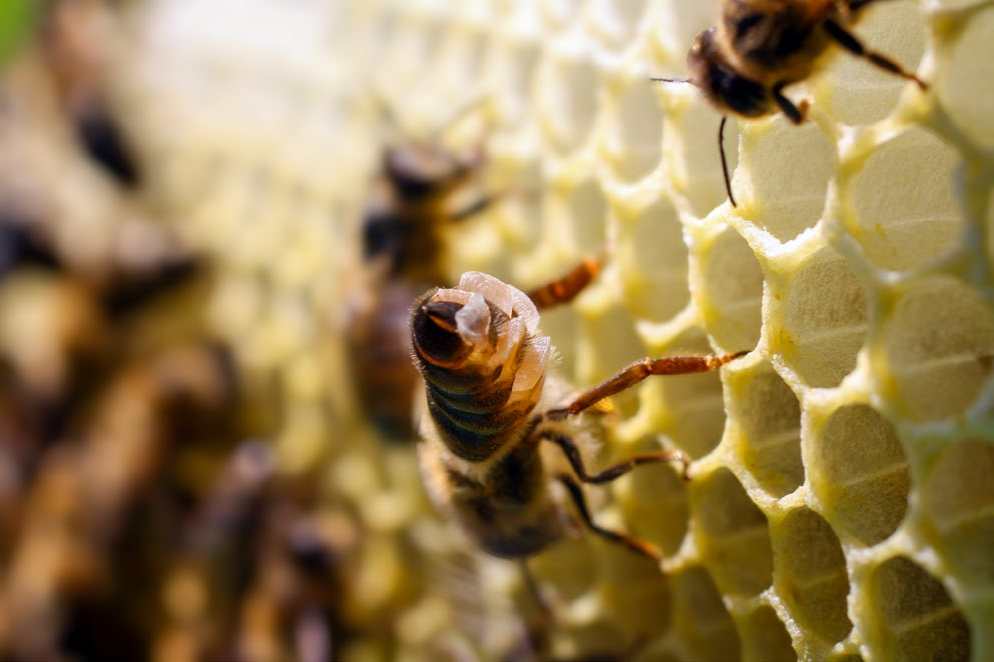
Wax‐producing worker honey bee ( *Apis mellifera* ) with freshly excreted wax scales visible at the abdominal wax glands during early comb construction. This physiological process is rarely observed in nature, as wax scales are typically removed and processed by nestmates within the concealed hive interior. The photograph was taken during the establishment phase of the mini‐hive experiment, when colonies were drawing out natural wax combs before experimental feeding treatments commenced. This fascinating depiction illustrates a central biological mechanism underlying colony development. Photograph by R. Odemer.

Building on our residue‐focused work (Odemer et al. 2023), the present study addresses this evidence gap by evaluating whether TMX seed treatments in sugar beet translate into measurable effects on honey bee colonies. We applied two complementary approaches: (i) monitoring full‐sized colonies placed at treated and untreated sugar beet sites to assess adult bee populations and brood development, and (ii) a parallel dietary exposure study using mini‐hives to assess individual worker survival under controlled conditions (Thompson et al. 2019) (Figure 1). The figure shows a worker honey bee producing freshly secreted wax scales—a rarely observed natural process—documented during the establishment of these mini‐hives as they built their own combs. By combining field monitoring with a controlled dietary exposure assay, we link verified exposure levels with both colony‐level endpoints and individual worker survival under real‐world agricultural conditions. The objectives of this study were to:

- Evaluate colony strength and brood production at two sugar beet sites in Germany;
- Assess individual worker survival in a dietary exposure study using mini‐hives;
- Link residue findings with colony‐ and individual‐level outcomes, thereby expanding on our previous results (Odemer et al. 2023).

## 2 Material and Methods

### 2.1 Experiment 1: Field Evaluation of Colony Development

#### 2.1.1 Study Sites and Test Organisms

The study focused on two main regions in Germany where emergency approval for thiamethoxam‐treated sugar beet was granted in 2021, covering a total of 34,700 ha in Lower Saxony (JKI) and 20,600 ha in Bavaria (VHH) (European Commission 2021).

The honey bee colonies were obtained from the respective institute's apiary. Ten successfully overwintered, queen‐right colonies were selected at the JKI site, and 18 colonies at the VHH site. Initial colony strength, including the number of adult bees and brood cells, was assessed using the Liebefeld method (Imdorf et al. 1987). Colonies were then randomly assigned to either thiamethoxam‐treated sugar beet (TMX) or control plots.

At each of the two JKI plots, five full‐sized *A. mellifera* colonies were installed, whereas six colonies were placed at each VHH plot. Control plots (JKI‐C and VHH‐C) without neonicotinoid treatment were located at least 2 km away from the treated plots before drilling. Plot sizes were 6.8 ha for JKI‐T and 15.3 ha and 13.6 ha for VHH‐T1 and VHH‐T2, respectively. Additional treated sugar beet fields were present within the foraging range of colonies at all treated plots, though the precise acreage was not quantified. In contrast, no other treated sugar beet fields were present within the foraging range of the control colonies. Maps of all five apiary locations (JKI C/T and VHH C/T1/T2) with a 2 km foraging radius are provided in Figures S2, S3. These maps serve as spatial orientation only; historical crop identity (including 2021 sugar beet boundaries) cannot be reliably reconstructed from current satellite imagery.

Residue analysis from the same 2021 field experiment confirmed that TMX and its metabolite CLO were present at very low levels in hive‐stored beebread (bee‐collected pollen) and in plant matrices collected adjacent to treated sugar beet fields (Odemer et al. 2023). In that study, beebread samples were taken from colonies at the JKI site at 7–10‐day intervals from drilling to harvest, while flowering weeds and bolting sugar beet shoots were sampled separately at both sites in late July and early August directly within or next to the treated fields. Although the palynological spectrum of beebread was not analyzed, TMX residues detected in this matrix were interpreted as originating mainly from neighboring weeds observed flowering in and around the sugar beet fields. TMX and CLO residues were also found in mud walls of *Osmia* nesting cavities placed within the foraging range of treated plots, whereas no residues of either compound were detected in any control samples. A consolidated overview of these residue data relevant to the present colony‐level analysis is provided in Table S4, and the full sampling effort of the 2021 monitoring campaign is summarized in Table S5. These findings verified exposure pathways beyond the sugar beet crop itself and established the exposure conditions under which the present colony‐level responses were assessed.

Regional weather conditions during the study period were obtained from the nearest stations of Germany's National Meteorological Service (DWD) (JKI: Magdeburg ID: 10361; VHH: Würzburg ID: 10655). Monthly mean temperature and precipitation summaries are provided in Table S6. Weather patterns in 2021 were broadly similar at both sites and did not deviate from normal seasonal conditions.

#### 2.1.2 Seed Treatment

Seeds were treated with Cruiser 600 FS (approval no. 006034–00, Syngenta Agro GmbH, Germany), a formulation containing 600 g of TMX per liter. The maximum application rate was 82.5 mL per ha, equivalent to a seed unit of 1.1 per ha or 49.5 g of TMX per ha (European Commission 2021). Exposure to TMX and its metabolite CLO was verified by residue analysis and reported in detail in Odemer et al. (2023).

#### 2.1.3 Colony Conditions

At the JKI site, colony conditions—including the number of adult bees and brood cells—were assessed seven times throughout the period from seed drilling (March 25) to harvest (September 20–30), covering 172 days. Utilizing the Liebefeld method, we tracked these parameters to monitor colony development over the entire growing season. In contrast, logistical constraints led to assessments being conducted three times at the VHH site, from drilling (March 27–31) to BBCH 31–39 of sugar beet, corresponding to rosette growth (May 25). This assessment period spanned 85 days.

#### 2.1.4 Statistical Analysis

To assess the impact of treatment and sampling time on honey bee colony parameters, we used negative‐binomial generalized linear mixed models (GLMMs). These models accounted for overdispersed count data and variability between replicates. Models were fitted using maximum likelihood (ML) estimation with the *nlminb* optimizer. Treatment (thiamethoxam‐treated versus control) and day after treatment (DAT) were included as fixed effects, whereas replicate was modeled as a random effect. The explanatory power of the models was evaluated using marginal *R* ^2^ (variance explained by fixed effects) and conditional *R* ^2^ (variance explained by both fixed and random effects).

All statistical analyses were conducted in R version 4.4.0 (R Core Team 2024). GLMMs were implemented using the glmmTMB package (Brooks et al. 2017; McGillycuddy et al. 2025). Visualization was carried out using ggplot2 (Wickham 2016). A significance level of *α* = 0.05 was applied for all tests.

##### 2.1.4.1 JKI Site

- *Bee population*: A negative‐binomial GLMM was applied to predict the number of adult bees with treatment and DAT as fixed effects and replicate as a random effect. The model showed a conditional *R* ^2^ of 0.57, with 16% of the variance attributed to fixed effects alone (marginal *R* ^2^ = 0.16).
- *Brood development*: For brood cell counts, a similar model was applied. This model exhibited strong explanatory power with a conditional *R* ^2^ of 0.72 and a marginal *R* ^2^ of 0.65.

##### 2.1.4.2 VHH Site

- *Bee population*: A negative‐binomial GLMM was used to predict the number of adult bees. The conditional *R* ^2^ for this model was 0.94, with 81% of the variance attributable to fixed effects (marginal *R* ^2^ = 0.81).
- *Brood development*: For brood cell counts, a similar model was applied, resulting in a conditional *R* ^2^ of 0.91 and a marginal *R* ^2^ of 0.86.

Residual diagnostics were conducted to verify model assumptions and fit. Full GLMM outputs for both sites are provided in Table S1.

### 2.2 Experiment 2: Worker Bee Survival in Mini‐Hives

A mini‐hive survival assay was re‐analyzed here with updated statistical methods because clothianidin was later detected in pollen and other matrices in the 2021 field experiment reported in Odemer et al. (2023). The verified exposure conditions motivate re‐evaluation of these survival data in the present context. This dataset complements Experiment 1 by extending the assessment from colony‐level outcomes to individual worker survival, thereby providing a more fine‐grained evaluation of CLO risks under the documented exposure scenario. The mini‐hive experiment itself was originally conducted in 2013 at the University of Hohenheim and made publicly available (Odemer and Odemer 2018). Although independent of the 2021 field experiment (Odemer et al. 2023), its design and endpoints are fully compatible with the present study, and the newly verified CLO exposure conditions provide a scientifically relevant framework for its re‐analysis.

#### 2.2.1 Mini‐Hive Setup

Experiment 2 was conducted from August to September using the “Kieler mating nuc” system, a Styrofoam box equipped with four top‐bars fitted with strips of beeswax foundation and an internal feeder, following the methodological description in Odemer and Odemer (2018). Twelve mini‐hives were established, each stocked with approximately 800 worker bees sourced from brood combs of two healthy donor colonies. Donors were verified to be free of clinical signs of brood diseases (e.g., American foulbrood, European foulbrood) and showed no visible symptoms of parasitic or viral infections (e.g., *Varroa destructor* , deformed wing virus).

Unmated sister queens were introduced into each mini‐hive. Colonies were held overnight in a dark and chilled room (15°C, approx. 16 h) before being placed outdoors at the institute apiary for natural mating. Within 5 weeks, all established colonies exhibited eggs, larvae, sealed brood, and newly built combs, confirming successful queen mating and colony establishment. Sister queens were used to minimize genetic variability within treatment groups, thereby reducing potential confounding maternal effects.

#### 2.2.2 Treatment and Exposure

Clothianidin (CLO) was selected as the test compound because it is the primary metabolite of thiamethoxam and exhibits comparable toxicity to honey bees (Thompson et al. 2021). Although the mini‐hive experiment was originally conducted in a different research context, it gains renewed relevance in light of the very low TMX and CLO residues detected during the same 2021 field experiment reported in Odemer et al. (2023). By re‐analyzing these data here, we link verified field exposure conditions with both colony‐level outcomes (Experiment 1) and individual worker survival (Experiment 2), thereby providing a more integrated assessment of risk under realistic agricultural conditions.

Clothianidin (99% purity, Dr. Ehrenstorfer GmbH) was prepared as a stock solution in purified water using ultrasonic treatment. Aliquots were diluted into sucrose feeding syrup (Apiinvert, Südzucker GmbH) to achieve a nominal concentration of 15 μg/kg. This concentration was chosen to remain below acute oral toxicity thresholds (Alkassab and Kirchner 2016) while representing environmentally realistic exposure levels. Later residue monitoring in sugar beet systems (Odemer et al. 2023) confirmed that this concentration corresponds well to the upper range of CLO residues in bee‐collected matrices, supporting the ecological relevance of the chosen dose. Control syrup was prepared with purified water only.

From the 12 initially established mini‐hives, 10 were selected and randomly assigned to treatment (*n* = 5) or control (*n* = 5). Each colony received 1.68 kg of syrup *ad libitum* over 26 consecutive days, corresponding to a total clothianidin intake of 25.2 μg per colony. Syrup uptake was confirmed by weighing feeders before and after feeding.

At the end of the exposure phase, one sealed brood comb per colony was removed and placed in a common incubator (34.5°C, 60% RH) for 24 h to standardize emergence conditions. From each treatment group, 100 newly emerged bees were randomly selected and individually marked with numbered opalith thorax plates. Marked bees from both groups (50 treated, 50 control) were simultaneously introduced into the two remaining mini‐hives (Col1 and Col2, from the original 12), creating mixed colonies that permitted side‐by‐side comparison under identical social and environmental conditions.

The 19‐day monitoring period was chosen because it spans the vulnerable post‐emergence phase during which workers transition from in‐hive tasks to orientation flights and, subsequently, foraging. This developmental window is known to be particularly sensitive to sublethal neonicotinoid effects on behavioral maturation and foraging performance (Colin et al. 2019). A schematic of the experimental workflow is shown in Figure S1.

#### 2.2.3 Monitoring of Mortality

Survival was monitored daily for 19 days, beginning 24 h after bee introduction. All combs and the interior of each mini‐hive were photographed under standardised light conditions in the early morning before major foraging activity. Marked bees were counted from photographs using the method of Odemer and Odemer (2018).

Recovery rate was defined as the proportion of marked bees observed at least once during the observation period. Rates ranged from 99%–100% across groups, confirming that hatching, marking, and introduction did not cause initial losses.

#### 2.2.4 Residue Analysis

To verify exposure, subsamples of the stock solution and prepared syrup were collected prior to feeding. On day 18 of the feeding period, several cells of stored food and stored pollen (beebread) were sampled from each mini‐hive. The subsamples from each colony were pooled to obtain one composite food sample and one composite pollen sample per colony. For residue confirmation, the colony‐level samples of each treatment group were then combined, yielding one pooled food sample and one pooled pollen sample per treatment group (four analytical samples in total).

Clothianidin (CLO) residues were analyzed by accredited external laboratories using LC–MS/MS as the primary quantification method. GC–MS was applied only as supplementary quality control for the syrup matrix, which reflects standard laboratory practice at the time. All analyses followed acetonitrile extraction and dispersive SPE cleanup according to the QuEChERS method (EN 15662:2009).

Matrix‐specific method performance data provided by the laboratories indicated that the limit of detection (LOD) and limit of quantification (LOQ) for feeding syrup and stored food were 1 μg/kg and 3 μg/kg, respectively (Eurofins Dr. Specht Laboratories, Hamburg), whereas for beebread, the LOD was 0.1 μg/kg and the LOQ 0.3 μg/kg (LUFA Speyer). Quantification was based on matrix‐matched calibration. No isotopically labeled internal standard was used for beebread analysis, which was consistent with routine CLO residue workflows at the time of analysis.

#### 2.2.5 Statistical Analysis

Worker survival was first analyzed by Kaplan–Meier survival analysis, with censored data assigned to bees not recovered at the end of the experiment. Group differences were tested using the log‐rank (Cox–Mantel) test.

To account for potential colony‐level effects, a mixed‐effects Cox proportional hazards model was fitted, with treatment (clothianidin vs. control) as a fixed effect and colony replicate (Col1 vs. Col2) as a random effect. This refinement goes beyond the original 2018 analysis, providing a more robust treatment of colony‐level variation. Model fit and proportional hazards assumptions were evaluated using Schoenfeld residuals.

All analyses were conducted in R version 4.4.0 (R Core Team 2024). Survival analyses were performed using the packages survival (Therneau 2024a; Therneau and Grambsch 2000) and coxme (Therneau 2024b). Visualizations were generated with the packages survminer (Kassambara et al. 2025) and ggplot2 (Wickham 2016). Statistical significance was set at *α* = 0.05. Hazard ratio estimates, confidence intervals, and diagnostics are provided in Table S2. Kaplan–Meier estimates and summary statistics are presented in Table S3.

## 3 Results

### 3.1 Experiment 1: Field Evaluation of Colony Development

#### 3.1.1 Colony Conditions

The honey bee colonies at both the JKI (Lower Saxony) and VHH (Bavaria) sites were assessed for colony strength and development over the growing season. The metrics included the number of adult bees and brood cells. Sampling dates are reported as days after treatment (DAT).

Figures display only interaction terms (treatment × DAT), whereas the text also reports the main temporal effects (DAT relative to baseline) to guide interpretation of seasonal growth.

##### 3.1.1.1 JKI Site

Colony conditions were monitored seven times from seed drilling to harvest (DAT0–DAT172).

**FIGURE 2:**
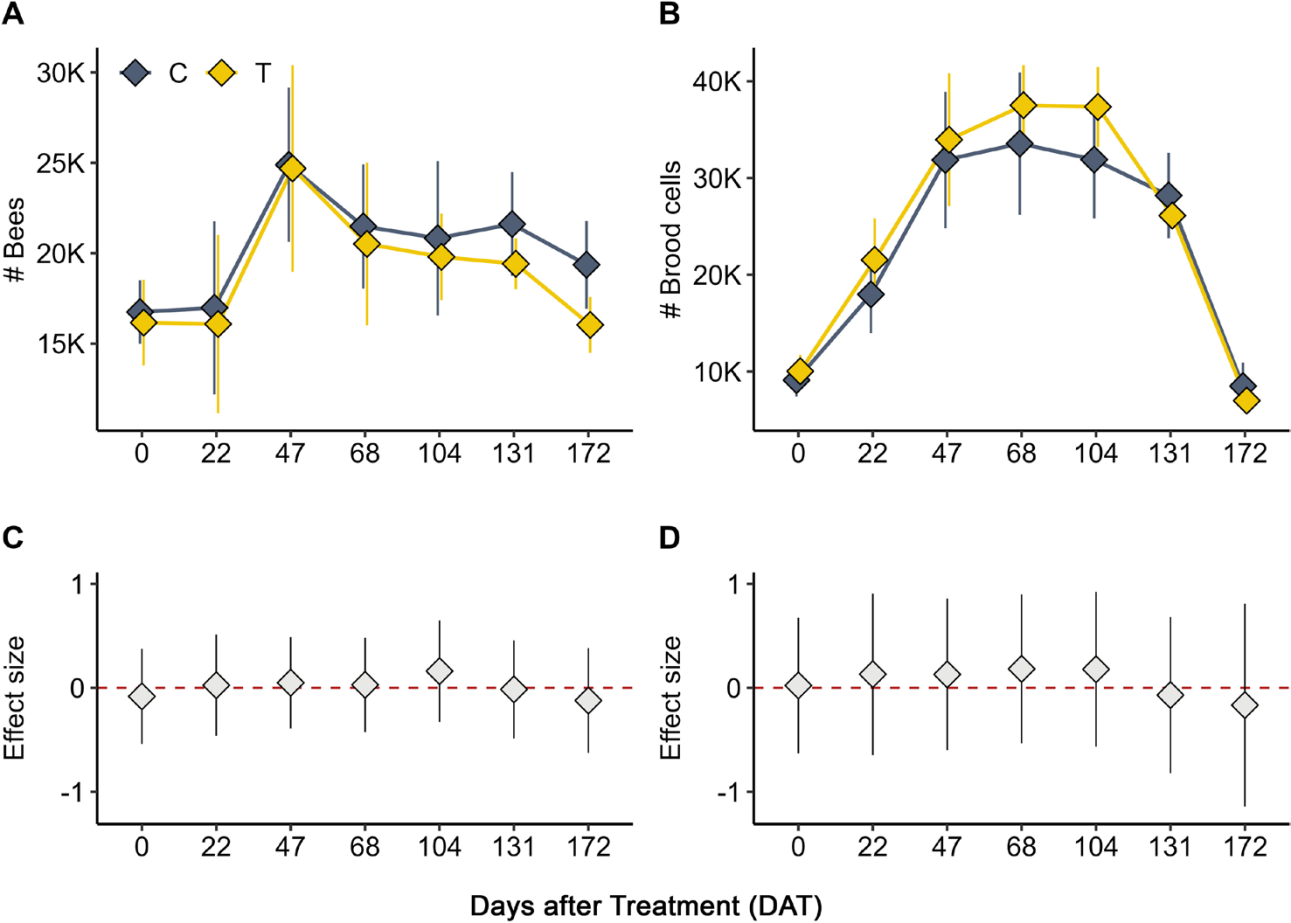
Overview of honey bee colony dynamics and treatment effects at the JKI site. (A) Mean number of adult bees over time (DAT) in treatment (T) and control (C) colonies, showing seasonal development. (B) Mean number of brood cells over time (DAT) in treatment (T) and control (C) colonies. (C) Effect sizes (IRR, treatment × DAT interaction terms) for the number of bees. (D) Effect sizes (IRR, treatment × DAT interaction terms) for the number of brood cells. Significant terms (*p* < 0.05) are marked with asterisks. Error bars = SEM. Baseline (DAT0) confirms no differences at study start.

*Bee population*: No significant treatment × DAT interactions were detected (e.g., DAT22: IRR = 1.03, *p* = 0.919; Figure 2C). Main temporal increases relative to baseline were observed across both groups (DAT47: IRR = 1.40, *p* = 0.035), consistent with seasonal population growth (Figure 2A). Fixed effects explained 16% of variance (marginal *R* ^2^ = 0.16), with 57% explained overall (conditional *R* ^2^ = 0.57).

*Brood development*: Significant temporal increases were detected (DAT47: IRR = 2.61, *p* < 0.001; DAT68: IRR = 2.83, *p* < 0.001; Figure 2B). No significant treatment × DAT interactions were found (Figure 2D). Fixed effects explained 65% of variance (marginal *R* ^2^ = 0.65), with 72% explained overall (conditional *R* ^2^ = 0.72).

##### 3.1.1.2 VHH Site

Colony conditions were evaluated three times (DAT0–DAT85).

**FIGURE 3:**
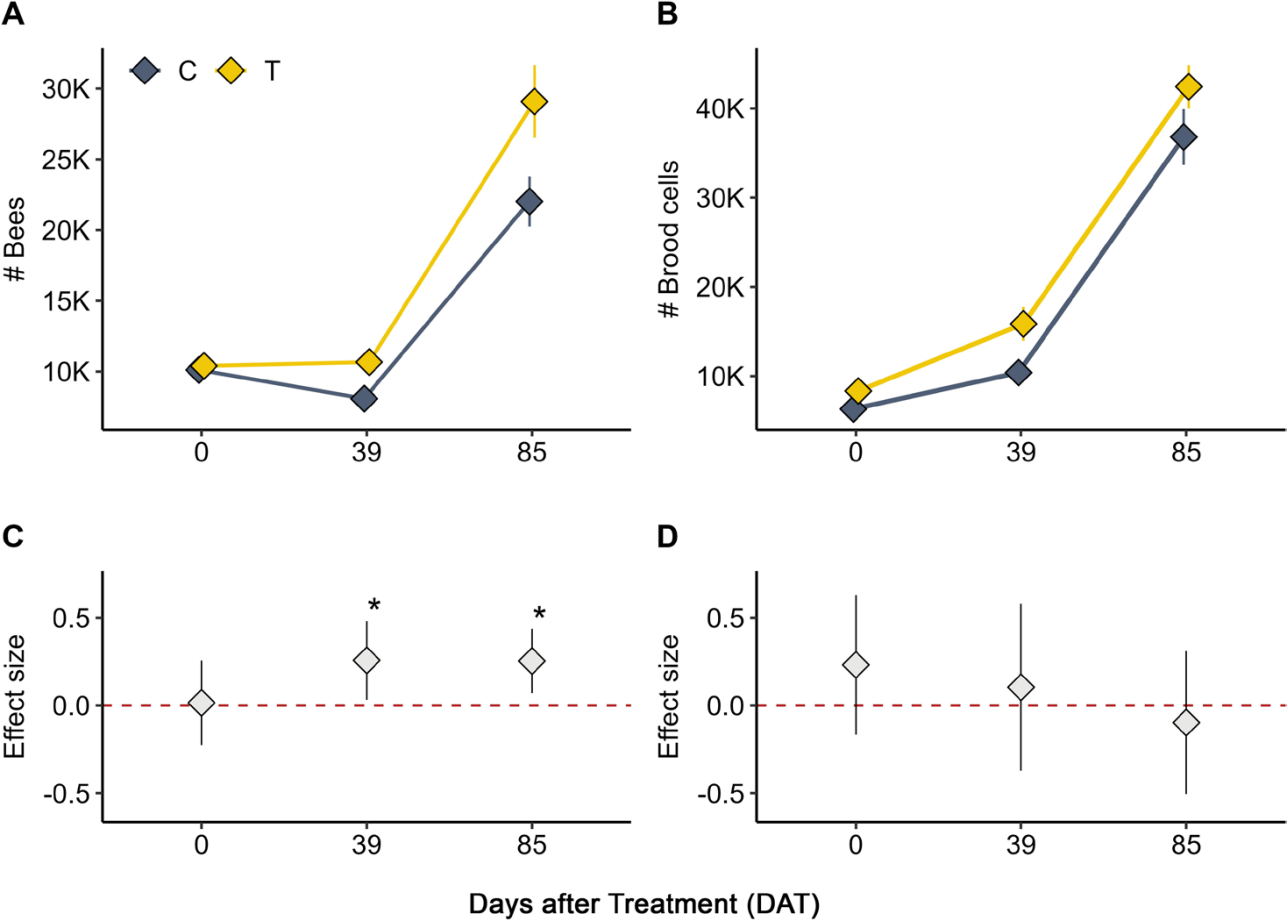
Overview of honey bee colony dynamics and treatment effects at the VHH site. (A) Mean number of adult bees over time (DAT) in treatment (T) and control (C) colonies, showing seasonal development. (B) Mean number of brood cells over time (DAT) in treatment (T) and control (C) colonies. (C) Effect sizes (IRR, treatment × DAT interaction terms) for the number of bees. Significant terms (*p* < 0.05) are marked with asterisks. (D) Effect sizes (IRR, treatment × DAT interaction terms) for the number of brood cells. Error bars = SEM. Baseline (DAT0) confirms no differences at study start.

*Bee population*: Significant treatment × DAT interactions were detected (DAT39: IRR = 1.29, *p* = 0.025; DAT85: IRR = 1.29, *p* = 0.007; Figure 3C). Main temporal increases relative to baseline were also evident (DAT85: IRR = 2.17, *p* < 0.001), consistent with seasonal colony growth (Figure 3A). The overall explanatory power was high (conditional *R* ^2^ = 0.94).

*Brood development*: Brood cell counts increased significantly over time (DAT39: IRR = 1.58, *p* = 0.026; DAT85: IRR = 5.45, *p* < 0.001; Figure 3B), whereas treatment × DAT interactions were not significant (Figure 3D). The model explained 87% of variance via fixed effects (marginal *R* ^2^ = 0.87), with 91% explained overall (conditional *R* ^2^ = 0.91).

### 3.2 Impacts of Thiamethoxam

Across sites, temporal effects (days after treatment, DAT) consistently explained most of the variation in colony metrics, reflecting seasonal growth in both bee and brood populations. At the JKI site, no significant interactions between treatment and DAT were detected, and treatment alone accounted for little variance compared with time (marginal *R* ^2^ = 0.16 for bees; 0.65 for brood). At the VHH site, two significant treatment × DAT interactions were observed for the bee population (DAT39: IRR = 1.29, *p* = 0.025; DAT85: IRR = 1.29, *p* = 0.007), whereas brood dynamics were driven by time alone. Thus, treatment‐related effects were only evident for adult bee numbers at the VHH site, whereas brood development and colony growth at both sites were largely explained by temporal progression.

### 3.3 Experiment 2: Worker Bee Survival in Mini‐Hives

**FIGURE 4:**
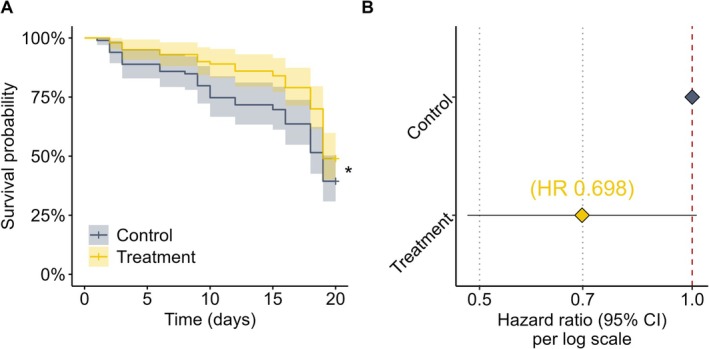
Survival analysis of honey bees under in‐hive clothianidin exposure. (A) Kaplan–Meier survival curves of newly emerged, individually marked workers originating from colonies receiving clothianidin‐treated (yellow) or control (gray) syrup during brood rearing. After emergence, marked bees were monitored for 19 days under identical conditions. Shaded areas indicate 95% confidence intervals. The log‐rank test showed a significant difference between the survival curves (*χ* ^2^ = 4.11, df = 1, *p* = 0.043); the asterisk in panel (A) denotes this significance level. (B) Hazard ratios from the mixed‐effects Cox model, including colony identity as a random effect. The estimated hazard ratio for clothianidin treatment was 0.698 (95% CI: 0.498–1.000, *p* = 0.060). The vertical dashed line marks HR = 1 (no effect).

Kaplan–Meier survival analysis indicated differences between the control and clothianidin treatment groups (Figure 4A). Survival probabilities in the control group steadily declined, with a final survival probability of 0.394 (95% CI: 0.309–0.503) by day 19. The clothianidin‐treated group showed a higher survival probability of 0.490 (95% CI: 0.401–0.598) at day 19. A log‐rank test detected a statistically significant difference in the survival distributions (*χ* ^2^ = 4.11, df = 1, *p* = 0.043).

The mixed‐effects Cox proportional hazards model additionally accounted for colony‐level variability. The random effect for replicate (Col1 vs. Col2) showed low variance (SD = 0.135), indicating limited inter‐colony differences. Fixed effects analysis estimated a hazard ratio of 0.698 for clothianidin treatment compared to control (95% CI: 0.498–1.000, *p* = 0.060), suggesting a slight tendency toward higher survival in treated bees (Figure 4B). Unlike the log‐rank test, this model did not identify a statistically significant treatment effect at the 5% level.

Model fit was evaluated using penalized log‐likelihood, corresponding information criteria are reported in Supplementary Table S3.

#### 3.3.1 Residue Analysis

The laboratory analysis confirmed the intended clothianidin concentration of 15 micrograms per kilogram (μg/kg) in the feeding syrup of Experiment 2. Residue analysis of stored food and pollen from the clothianidin‐treated mini‐hives revealed measurable residues ranging from approximately 2 to 6 μg/kg in stored food and 1.79 μg/kg in stored pollen after 18 days. Residue levels in the untreated control hives were below the analytical reporting limit and are therefore reported as <LOQ in Table 1. These findings verify that clothianidin residues accumulated in the treated mini‐hives while controls remained below quantifiable levels. Values shown in Table 1 originate from treatment‐level pooled composites (see Materials & Methods for sampling and pooling details).

**TABLE 1:** Residue analysis of feeding syrup and pooled samples from stored food and pollen combs in control and clothianidin‐treated mini‐hives after 18 days of feeding (LC–MS/MS, limit of quantification: 3 μg/kg for food, 0.3 μg/kg for pollen).

Sample type	Control	Clothianidin treatment
Stock solution	—	15,000 µg/kg
Feeding syrup	<LOQ	15 μg/kg
Stored food	<LOQ	6 μg/kg
Stored pollen	<LOQ	1.79 μg/kg

## 4 Discussion

In 2021, Germany approved the emergency use of thiamethoxam–treated sugar beet seeds, despite the EU ban on neonicotinoids due to pollinator risks (Odemer et al. 2023). This underscores the need for field studies that evaluate pesticide impacts on honey bee colonies under realistic agricultural conditions, where variables such as forage diversity, pesticide dilution, and colony resilience can critically shape outcomes (Thompson et al. 2021; Alberoni et al. 2021; Wueppenhorst, Alkassab, Beims, Ernst, et al. 2024). Honey bees remain central to pollination services and biodiversity, making it vital to assess whether seed treatments in non‐flowering crops pose risks that outweigh their agricultural benefits (Samson‐Robert et al. 2017).

### 4.1 Colony‐Level Outcomes Under Field Conditions

In Experiment 1, across two geographically distinct sites, we detected no consistent adverse effects of TMX treatment on colony strength or brood development. At the VHH site, treatment × time interactions occasionally coincided with larger adult bee numbers in treated colonies, whereas at JKI, a late‐season contrast suggested approximately 20% fewer adult bees compared to controls. Although this reduction numerically exceeds the ≤ 10% colony size threshold defined as a specific protection goal in the updated EFSA Bee Guidance Document (EFSA 2023), the effect did not persist across sites and was not accompanied by consistent brood differences. This illustrates both the buffering capacity of colonies and the high context dependence of field outcomes, as also observed in other multi‐site neonicotinoid studies (Woodcock et al. 2017; Thompson et al. 2021; Flores et al. 2021).

Our results support previous findings that colonies can compensate for stress through mechanisms such as brood replacement and altered resource allocation (Overmyer et al. 2018; Schott et al. 2021). This buffering may explain why statistically significant treatment effects were rare, a pattern also recognized in the debate around field trials, where effect sizes, not only *p*‐values, should be emphasized (Woodcock et al. 2018).

Importantly, the residue analysis conducted as part of the same 2021 field experiment (Odemer et al. 2023) found no evidence of acute or chronic risk to honey bees when assessed using the U.S. EPA BeeREX model. Although residues were occasionally detected in pollen and weeds, nectar and honey samples were consistently below detection (see also Woodcock et al. 2021), supporting the conclusion that exposure levels in sugar beet systems are generally negligible. These findings are consistent with the large‐scale multi‐country study of Thompson et al. (2021), which similarly reported that residues in pollen and nectar of succeeding crops following TMX‐treated sugar beet were at or below quantifiable levels in nearly all samples. Comparable results were also obtained in a three‐year Spanish field trial with sunflowers (Flores et al. 2021), where only transient early reductions in adult bees and brood were observed, but no long‐term treatment effects persisted. Taken together, the absence of consistent treatment effects in our endpoints is therefore most plausibly explained by limited exposure, with colony buffering mechanisms playing a secondary role in stabilizing outcomes.

### 4.2 Worker Survival and Sublethal Effects

Experiment 2, which provided complementary survival data, revealed no statistically significant difference in worker longevity between treatment groups when analyzed with the mixed‐effects Cox model, which accounted for colony‐level variation (hazard ratio ≈0.70, 95% CI: 0.50–1.00, *p* = 0.06). This model provides a stronger basis for inference than the Kaplan–Meier log‐rank test, which had suggested a weak difference. We therefore conclude that the survival assay indicates no adverse effect of clothianidin exposure under the tested conditions. Residues measured in stored food and pollen from the mini‐hives (≈2–6 μg/kg) closely overlapped the upper range of TMX + CLO residues detected in bee‐collected matrices during the 2021 field experiment (Odemer et al. 2023). This alignment indicates that the mini‐hive exposure realistically reflected upper‐bound field exposure conditions, strengthening the link between the survival assay and the colony‐level monitoring.

Nonetheless, such outcomes can be considered within broader mechanistic frameworks. Apparent survival increases under low‐level exposure have sometimes been linked to hormesis concepts, where stressors may stimulate compensatory performance traits (Cutler and Rix 2015; Rix and Cutler 2020; Cutler et al. 2022). Similar cross‐stressor interactions have been reported in bees, for example, improved tolerance to heat stress under sublethal neonicotinoid exposure (Colgan et al. 2020). Although such mechanisms remain speculative in the context of our findings, they illustrate the potential for non‐linear responses that may help explain occasional positive trends in survival data.

### 4.3 Role of Seasonality and Environment

Significant seasonal growth trends were observed at both sites, reflecting natural colony development under favorable forage conditions (Démares et al. 2018; Siede et al. 2018). Nutritional quality and access to diverse pollen are known to mitigate pesticide effects (Castle et al. 2022, 2023) and may have further supported resilience in our colonies. Environmental variability also explains why field outcomes often diverge from laboratory findings (Henry et al. 2015; Tsvetkov and Zayed 2021). Genetic background may further contribute, as patriline‐level variation in CYP9Q haplotypes influences honey bee survival under neonicotinoid exposure (Tsvetkov et al. 2023), consistent with molecular evidence that CYP9Q detoxification enzymes shape species‐specific sensitivity to these compounds (Manjon et al. 2018). Under field conditions, dynamic foraging and trophallactic filtering may dilute residues before they reach sensitive life stages (Wueppenhorst, Alkassab, Beims, Bischoff, et al. 2024). This may account for the lack of consistent colony‐level effects, despite reports of neurobehavioral changes in controlled exposure studies (EFSA 2013; Samson‐Robert et al. 2017).

### 4.4 Limitations

As in other field experiments, replication was constrained by logistical factors, and colonies within a site shared environmental conditions, introducing pseudoreplication (Bailey and Greenwood 2018). Although no consistent treatment effects were detected, limited replication means that smaller effect sizes below our detection threshold cannot be excluded. Forage availability and weather likely influenced outcomes, and background exposure to agrochemicals other than thiamethoxam/clothianidin cannot be ruled out (Woodcock et al. 2018). However, residue analyses from the same 2021 field experiment detected no thiamethoxam or clothianidin in any control samples, indicating that exposure to these actives did not confound treatment–control comparisons. Finally, our endpoints focused on colony strength and survival, whereas more granular behavioral and physiological measures could reveal sublethal effects not captured here.

### 4.5 Outlook

Future studies should address these limitations by harmonizing sampling across sites, randomizing colony placement, and extending monitoring to overwintering phases (Tosi et al. 2022; Sabo et al. 2024). Evidence from Canadian field trials indicates that only unrealistically high doses of thiamethoxam reduced overwinter survival, whereas field‐realistic exposures had no consistent effect (Wood et al. 2020). Advanced sensor‐based monitoring, already shown to detect subtle colony‐level changes under sublethal pesticide exposure (Meikle and Weiss 2017), may further improve detection of behavioral impairments (Odemer et al. 2024; Borlinghaus et al. 2024; Wang et al. 2024).

## 5 Conclusion

This study shows that thiamethoxam‐treated sugar beet did not cause consistent adverse effects on honey bee colonies under the tested field conditions. Although a late‐season reduction in adult bees was observed at one site, this was not replicated elsewhere, and survival assays showed no significant adverse impacts on worker longevity. The main contribution of this work is the integration of colony‐level monitoring with re‐analyzed survival data, interpreted in the context of previously documented residue exposure from the same 2021 field experiment. This provides a more comprehensive and realistic assessment of risk than single approaches alone.

These results support the view that exposure pathways from sugar beet seed treatments are minimal, with observed variability most likely reflecting natural environmental and seasonal factors rather than treatment effects. Nonetheless, limited replication and site differences highlight the need for long‐term, multi‐site studies that include both colony‐level and behavioral endpoints.

Overall, our findings strengthen the evidence that thiamethoxam use in sugar beet poses limited risk to honey bee colonies under practical agricultural conditions while underlining the importance of robust, field‐based evaluations for pollinator risk assessment.

## Author Contributions

**Richard Odemer:** conceptualization (equal), data curation (lead), formal analysis (lead), investigation (equal), methodology (equal), software (lead), visualization (lead), writing – original draft (lead), writing – review and editing (lead). **Stefan Berg:** conceptualization (equal), funding acquisition (equal), investigation (equal), methodology (equal), project administration (equal), resources (equal), writing – review and editing (supporting). **Jens Pistorius:** resources (equal), writing – review and editing (supporting). **Ingrid Illies:** conceptualization (equal), funding acquisition (equal), investigation (equal), methodology (equal), project administration (equal), writing – review and editing (supporting).

## Funding

This study was conducted within the institutional research framework of the Julius Kühn‐Institut (JKI), Federal Research Centre for Cultivated Plants. The fieldwork at the VHH site was supported by the Bavarian State Ministry of Food, Agriculture and Forestry. No external commercial funding was received for this research. Mention of brand names or commercial products in this publication is solely for the purpose of providing specific information and does not constitute endorsement by the JKI.

## Ethics Statement

All applicable international, national, and/or institutional guidelines for the careful handling and use of animals were followed. In the context of the article, none of the authors conducted studies with human subjects.

## Conflicts of Interest

The authors declare no conflicts of interest.

## Supporting information


**Data S1:** ece372767‐sup‐0001‐DataS1.docx.

## Data Availability

The data that support the findings of this study are available in the “Open Science Framework” under https://doi.org/10.17605/OSF.IO/B4DX8.
