# Supplementary material for: Honey Bee Colony Health in Thiamethoxam‐Treated Sugar Beet Fields: A Field‐Based Case Study
Source: Ecol Evol. 2025 Dec 23;15(12):e72767. doi: 10.1002/ece3.72767 (PMC12723444; doi:10.1002/ece3.72767)

Honey Bee Colony Health in Thiamethoxam-Treated Sugar Beet Fields: A Field-Based Case Study

Richard Odemer^1*^, Stefan Berg^2^, Jens Pistorius^1^, Ingrid Illies^2^

^1^Julius Kühn-Institut (JKI) – Federal Research Centre for Cultivated Plants, Institute for Bee Protection, Braunschweig, Germany

^2^Bavarian State Institute for Viticulture and Horticulture, Institute for Bee Research and Beekeeping (IBI), Veitshöchheim, Germany

*Corresponding Author: richard.odemer@julius-kuehn.de

SUPPLEMENTARY MATERIAL

**Supplementary Table S1.** Generalized Linear Mixed Model (GLMM) Outputs: Colony Development (Experiment 1)

**1. JKI Site**

**1.1. Adult Bee Numbers**

- Significant temporal effect: DAT47 showed an increase (IRR = 1.40, p = 0.035).
- No main effect of treatment and no significant interactions.
- **Random effects:** ICC = 0.48
- **Model fit:** Marginal R² = 0.159; Conditional R² = 0.565


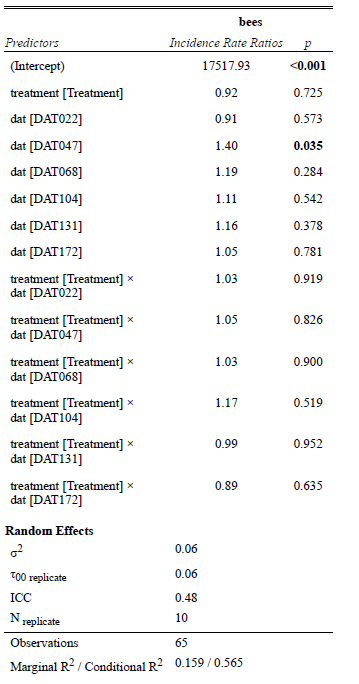


**1.2. Brood Cell Numbers**

- Strong temporal effects (DAT47–DAT131 all significant; DAT22 marginal, DAT172 not significant).
- No treatment or interaction effects.
- **Random effects:** ICC = 0.20
- **Model fit:** Marginal R² = 0.650; Conditional R² = 0.719


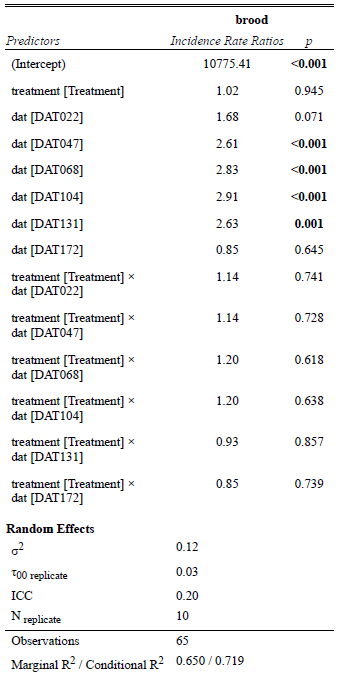


**2. VHH Site**

**2.1. Adult Bee Numbers**

- Strong temporal effects.
- Significant positive treatment × DAT interactions at DAT39 (p = 0.025) and DAT85 (p = 0.007), indicating higher adult bee numbers in treated colonies at these time points.
- **Random effects:** ICC = 0.69
- **Model fit:** Marginal R² = 0.813; Conditional R² = 0.943


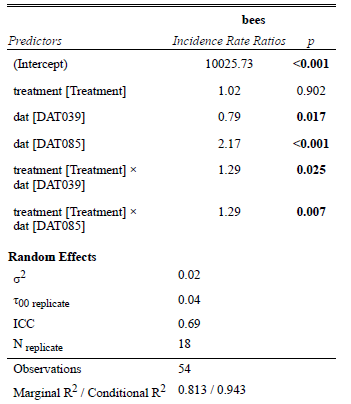


**2.2. Brood Cell Numbers**

- Temporal effects significant at DAT39 (p = 0.026) and DAT85 (p < 0.001).
- No treatment or interaction effects.
- **Random effects:** ICC = 0.29
- **Model fit:** Marginal R² = 0.867; Conditional R² = 0.905


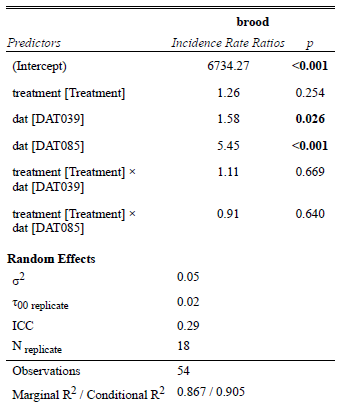


**Supplementary Table S2.** Summary of the mixed-effects Cox proportional hazards model (Experiment 2)

• Slightly increased survival in treated bees, but hazard ratios in Tab. S3 not biologically meaningful.

• No evidence of treatment-related reduction in survival.


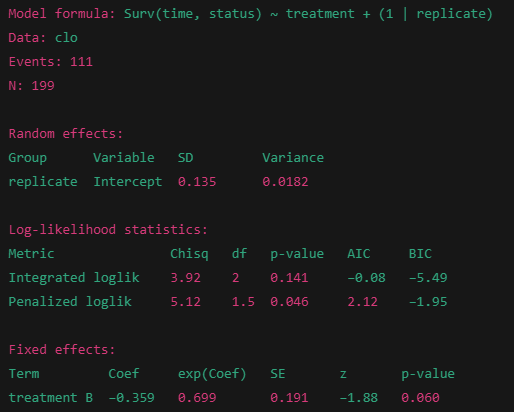


**Supplementary Table S3.** Kaplan–Meier survival estimates and Log-Rank test (Experiment 2)

• Survival curves differed weakly between treatment and control groups, consistent with the Cox model.

• No indication of biologically relevant adverse effects of treatment.


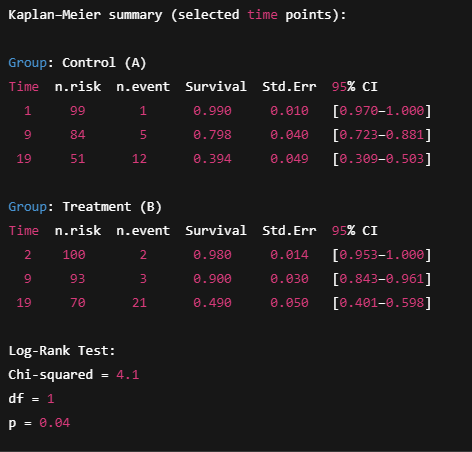


**Supplementary Table S4.** Summary of thiamethoxam (TMX), clothianidin (CLO), and total TMX + CLO residues detected in matrices collected from the JKI (Lower Saxony) and VHH (Bavaria) field sites during the 2021 sugar beet experiment. Only matrices relevant to the honey bee colonies monitored in the present study are included. Values represent concentration ranges (mg/kg) among treated samples. Control samples were consistently below LOQ. Bee-relevant matrices comprise nectar, honey crop, beebread, and dead bees. Weed and beet-shoot samples represent flowering weed species and bolting beet plants within the foraging range. Osmia mud walls were analysed within the same monitoring campaign but do not correspond to honey bee colony endpoints.

| Matrix | n (Control) | n (Treated) | Positive (Control) | Positive (Treated) | TMX range (µg/kg) | CLO range (µg/kg) | Total TMX + CLO range (µg/kg) | Sites |
| --- | --- | --- | --- | --- | --- | --- | --- | --- |
| Nectar / honey crop | 17 | 7 | 0 | 0 | <LOQ | <LOQ | <LOQ | JKI |
| Beebread | 13 | 3 | 0 | 3 | 0.273–0.466 | <LOQ | 0.273–0.466 | JKI |
| Dead bees | 15 | 6 | 0 | 0 | <LOQ | <LOQ | <LOQ | VHH |
| Weeds + shoots | 0 | 16 | – | 16 | 0–12.38 | 0.16–6.19 | 0.34–17.68 | JKI + VHH |
| Beet leaves (BBCH 33) | 1 | 1 | 1 (<LOQ) | 1 | 56.98 | 38.21 | 95.19 | JKI |
| Beet leaves (BBCH 49) | 1 | 1 | 0 | 1 | 0.27 | 2.44 | 2.72 | JKI |
| Beet seeds | 1 | 1 | 0 | 1 | 154,000 | <LOQ | 154,000 | JKI |
| Osmia mud walls* | 1 | 1 | 0 | 1 | 0.995 | <LOQ | 0.995 | JKI |

* Osmia matrices were collected as part of the same residue monitoring campaign but are not directly related to honey bee colony endpoints; included for transparency.

**Supplementary Table S5.** Overview of all TMX+CLO analyses from the 2021 sugar beet residue monitoring used to contextualize the present colony-level study. Sample numbers (n) in Table S4 refer to biological samples. Some plant samples were analytically split into technical subsamples (B1/B2, S1/S2). These appear separately in the raw data but count as one biological sample in this table, following the reporting structure of Odemer et al. (2023).

| Site | Matrix | Control (n) | Treated (n) | Notes |
| --- | --- | --- | --- | --- |
| JKI | Nectar/honey | 17 | 17 | no detections |
|  | Beebread/pollen | 13 | 10 | 3 positives |
|  | Dead bees | 0 | 0 | not sampled |
|  | Weeds | – | 8 | all positives |
|  | Shoots | – | 3 | all positives |
| VHH | Nectar/honey | 0 | 0 | not sampled |
|  | Beebread/pollen | 0 | 0 | not sampled |
|  | **Dead bees** | 8 | 15 | no detections |
|  | **Weeds** | – | 4 | all positives |
|  | **Shoots** | – | 2 | all positives |
| HOH | Pollen / Bees / Dead bees | – | – | *not part of present MS* |

Note: **Clarification on sample numbers (n) reported in Tables S4 and S5**

Table S5 lists all biological samples collected in 2021 (full design), whereas Table S4 includes only the subset of samples with relevant analytical information (e.g. quantifiable residues or matrices directly used in this study). Therefore, n-values differ between both tables by design.

**Supplementary Table S6.** Monthly mean temperature and precipitation for the study period (April–September 2021).
JKI = Magdeburg (MGDB) and VHH = Würzburg (WZBG), based on daily DWD KX climate data (values rounded).

| Month | Station | Mean Temperature (°C) | SD Temperature (°C) | Total Precipitation (mm) |
| --- | --- | --- | --- | --- |
| April | JKI (MGDB) | 7.0 | 2.77 | 25.9 |
|  | VHH (WZBG) | 7.5 | 3.61 | 17.6 |
| May | JKI (MGDB) | 12.3 | 3.10 | 53.3 |
|  | VHH (WZBG) | 11.4 | 2.69 | 69.8 |
| June | JKI (MGDB) | 20.5 | 2.98 | 59.1 |
|  | VHH (WZBG) | 20.1 | 2.69 | 111.0 |
| July | JKI (MGDB) | 20.3 | 2.10 | 22.2 |
|  | VHH (WZBG) | 18.8 | 1.65 | 130.0 |
| August | JKI (MGDB) | 17.8 | 2.13 | 94.3 |
|  | VHH (WZBG) | 17.3 | 2.51 | 80.4 |
| September | JKI (MGDB) | 16.2 | 2.46 | 30.2 |
|  | VHH (WZBG) | 16.4 | 2.68 | 6.0 |

**Weather data acquisition and processing**

Daily temperature (TMK) and precipitation (RSK) data were obtained from the Deutscher Wetterdienst Climate Data Center (DWD-CDC) in KX format. For the JKI site, the Magdeburg station (ID 10361) was used; for the VHH site, the Würzburg station (ID 10655). TMK (characters 79–82) and RSK (characters 246–249) were extracted following the official DWD field specifications. Implausible values were removed based on DWD conventions. Data were aggregated to monthly mean temperature and monthly precipitation totals and rounded for reporting.

**Citation:**
*DWD Climate Data Center (CDC): Daily station observations (KX-format)*, last accessed: 2025-11-25.

**Supplementary Figure S1.**

**Figure S1.** Experimental setup summarizing exposure, emergence, marking, and monitoring phases from Experiment 2.

Twelve mini-hives (“Kieler mating nucs”) were initially established with ~800 workers and unmated sister queens. After queen mating and colony establishment, ten colonies were randomly assigned to clothianidin-treated (n = 5) or control (n = 5) groups. Treated colonies received sucrose syrup containing 15 µg/kg clothianidin for 26 consecutive days, while control colonies received untreated syrup. At the end of the exposure phase, one sealed brood comb per colony was incubated for 24 h (34.5 °C, 60% RH), and 100 newly emerged workers from each treatment group were individually marked. Marked workers (50 treated and 50 control per hive) were introduced into the two remaining neutral recipient mini-hives to allow side-by-side comparison under identical social and environmental conditions. Worker mortality was recorded daily over a 19-day monitoring period. Residue confirmation was performed on feeding syrup, stored food, and stored pollen as described in the Methods.

**Supplementary Figure S2 (A-B).** Showing JKI the colony locations adjacent to the respective sugar beet fields and a 2 km foraging radius (green = control, red = treated).

**
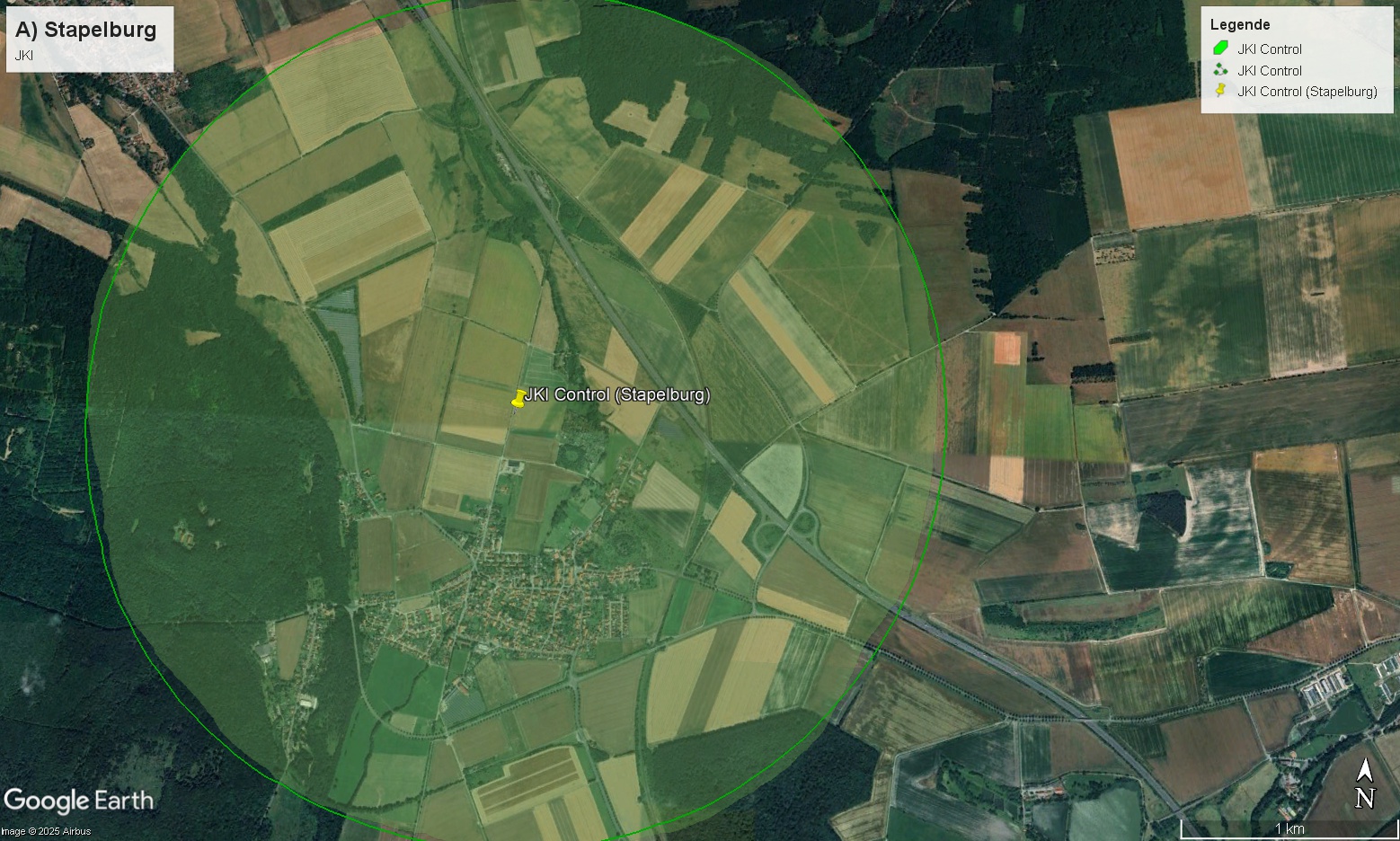
**

**
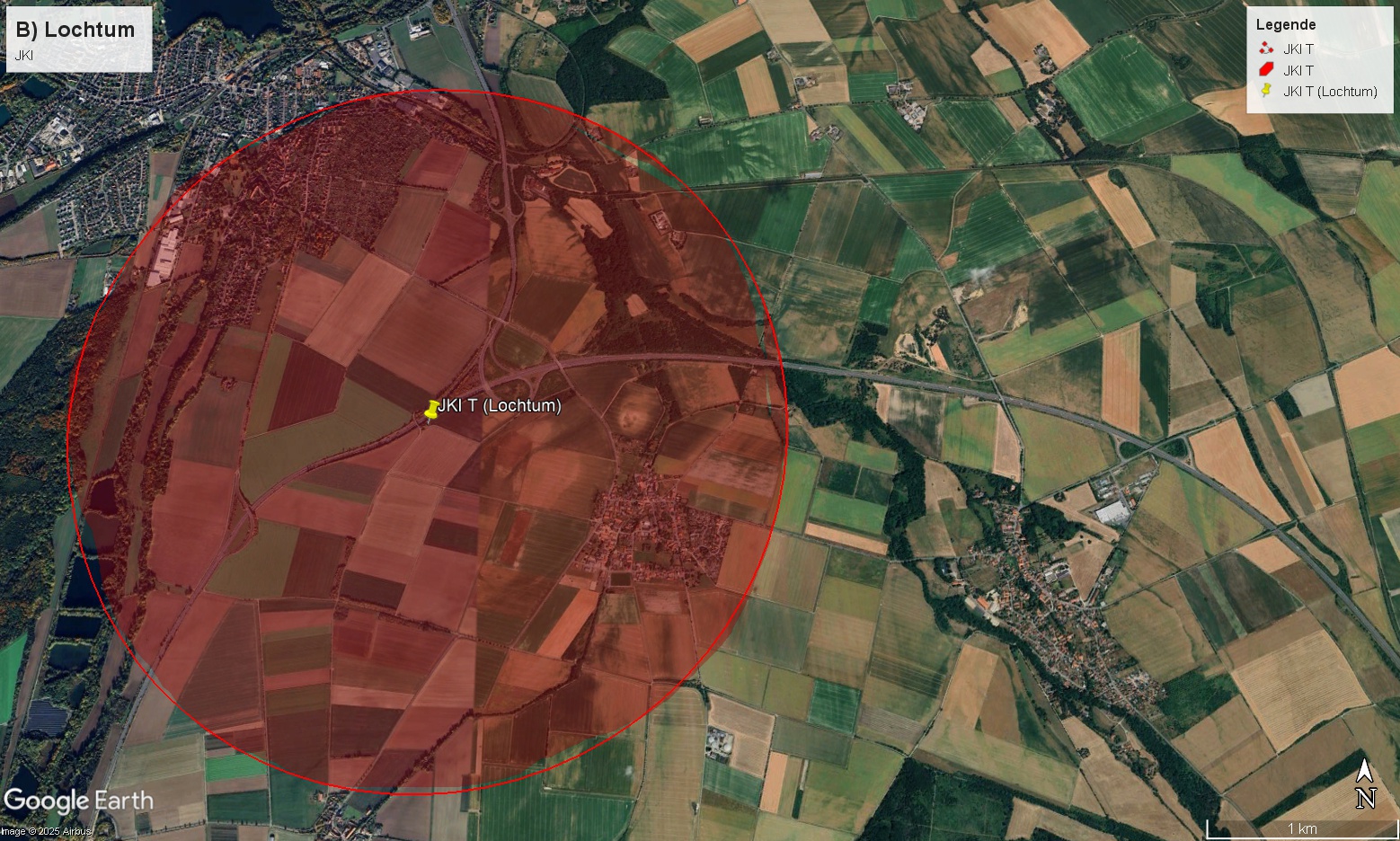
**

**Supplementary Figure S3 (A-C).** Showing the VHH colony locations adjacent to the respective sugar beet fields and a 2 km foraging radius (green = control, red = treated).


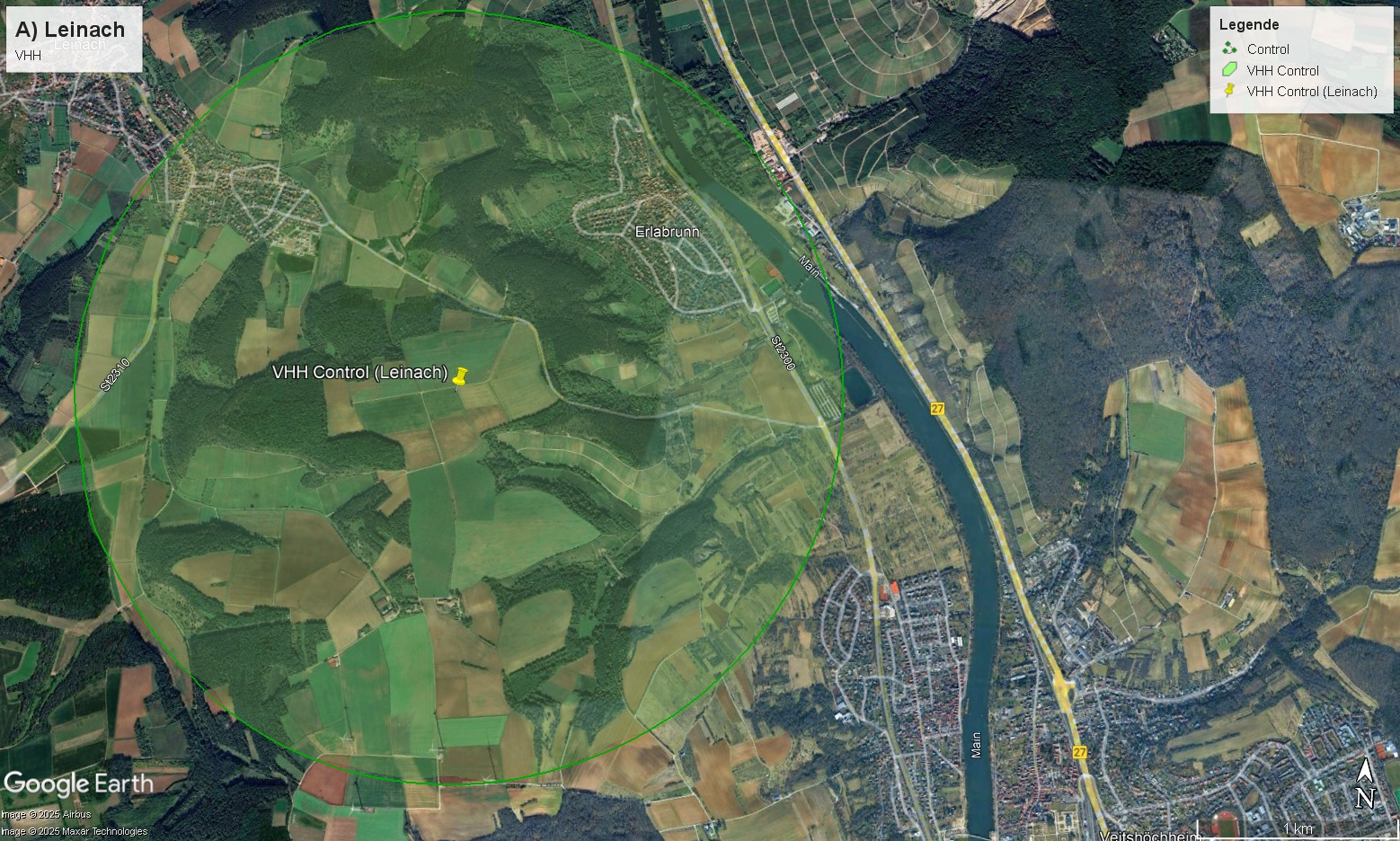


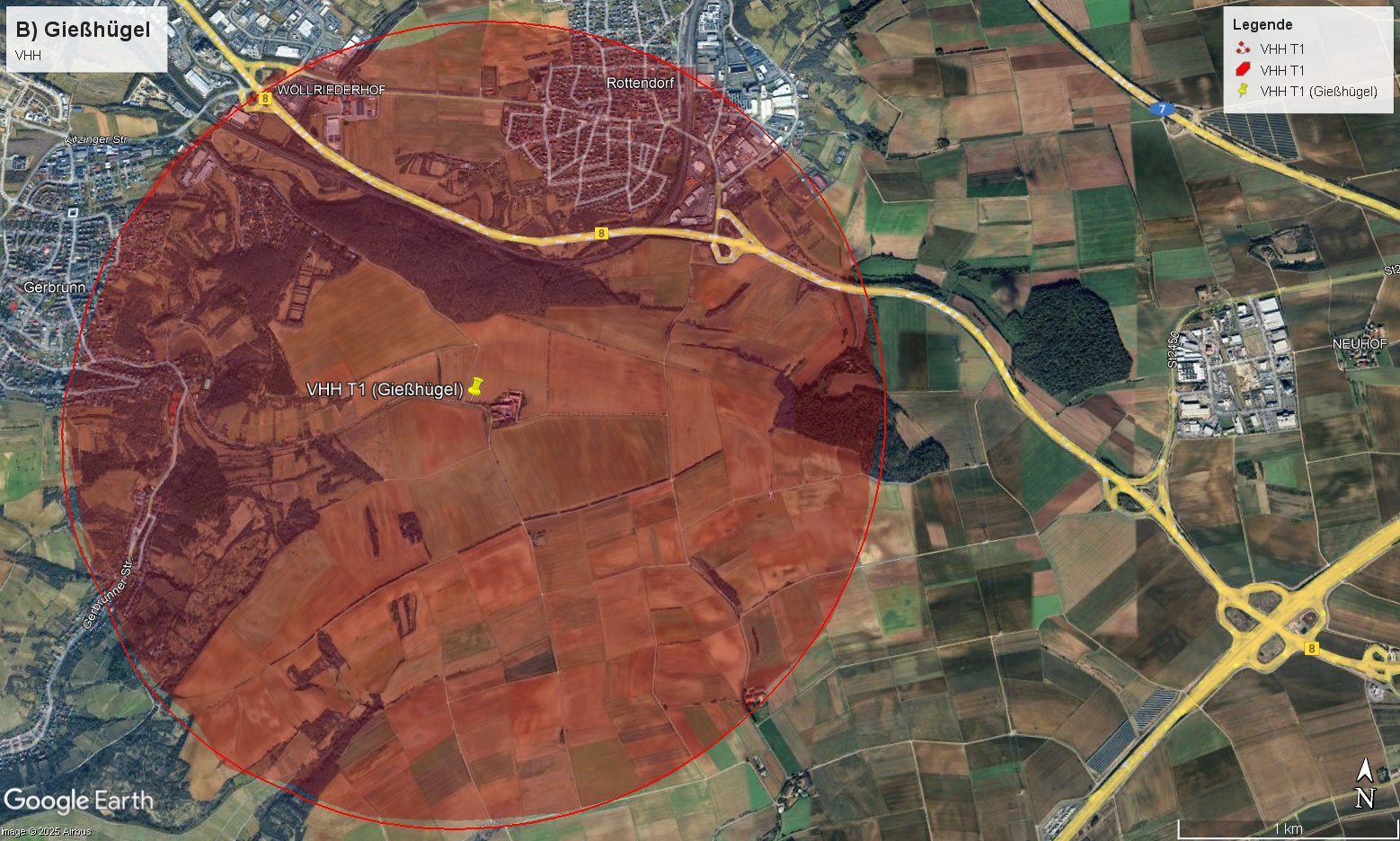


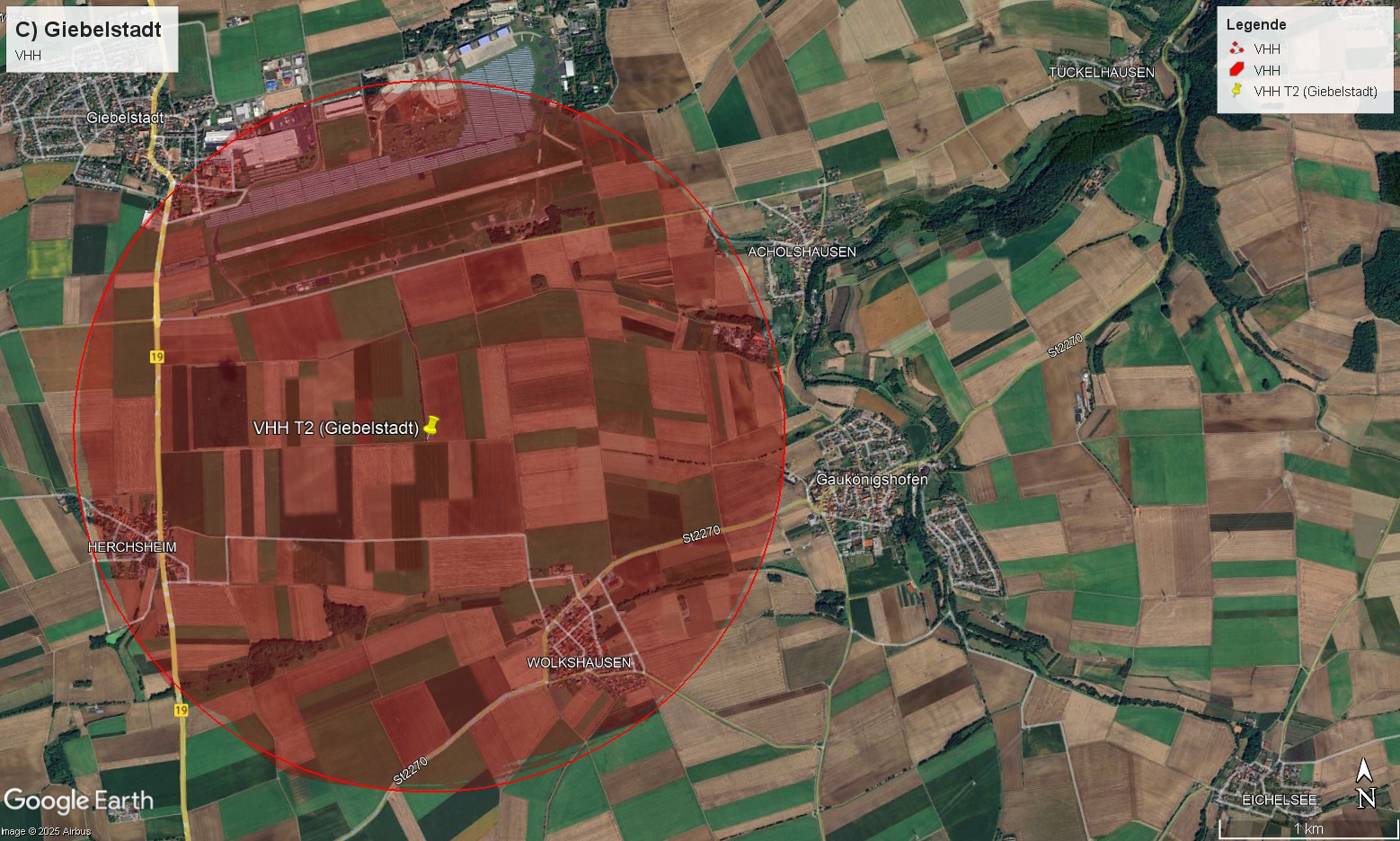

Supplement: Supplementary file 1 — Data S1: ece372767‐sup‐0001‐DataS1.docx. [file ECE3-15-e72767-s001.docx]
